# Targeted next-generation sequencing identifies novel variants in candidate genes for Parkinson’s disease in Black South African and Nigerian patients

**DOI:** 10.1186/s12881-020-0953-1

**Published:** 2020-02-04

**Authors:** Oluwafemi G. Oluwole, Helena Kuivaniemi, Shameemah Abrahams, William L. Haylett, Alvera A. Vorster, Carel J. van Heerden, Colin P. Kenyon, David L. Tabb, Michael B. Fawale, Taofiki A. Sunmonu, Abiodun Ajose, Matthew O. Olaogun, Anastasia C. Rossouw, Ludo S. van Hillegondsberg, Jonathan Carr, Owen A. Ross, Morenikeji A. Komolafe, Gerard Tromp, Soraya Bardien

**Affiliations:** 1 Division of Molecular Biology and Human Genetics, Department of Biomedical Sciences, Stellenbosch University, Cape Town, South Africa; 2 Division of Endocrinology, Department of Medicine, Faculty of Medicine and Health Sciences, Stellenbosch University, Cape Town, South Africa; 3 DNA Sequencing Unit, Central Analytical Facility, Stellenbosch University, Stellenbosch, South Africa; 4 Bioinformatics Unit, South African Tuberculosis Bioinformatics Initiative, Stellenbosch University, Cape Town, South Africa; 5 DST–NRF Centre of Excellence for Biomedical Tuberculosis Research, Stellenbosch University, Cape Town, South Africa; 6 South African Medical Research Council Centre for Tuberculosis Research, Stellenbosch University, Cape Town, South Africa; 7 Centre for Bioinformatics and Computational Biology, Stellenbosch University, Stellenbosch, South Africa; 8 Neurology Unit, Department of Medicine, College of Health Sciences, Obafemi Awolowo University, Ile-Ife, Nigeria; 9 Neurology Unit, Department of Medicine, Federal Medical Centre, Owo, Nigeria; 10 Department of Chemical Pathology, College of Health Sciences, Obafemi Awolowo University, Ile-Ife, Nigeria; 11 Department of Medical Rehabilitation, College of Health Sciences, Obafemi Awolowo University, Ile-Ife, Nigeria; 12 Division of Neurology, Department of Medicine, Faculty of Health Sciences, Walter Sisulu University, East London, South Africa; 13 Division of Neurology, Department of Medicine, Faculty of Medicine and Health Sciences, Stellenbosch University, Cape Town, South Africa; 14 Department of Neuroscience, Mayo Clinic, Jacksonville, Florida USA; 15 Department of Clinical Genomics, Mayo Clinic College of Medicine, Jacksonville, Florida USA

**Keywords:** Parkinson’s disease, Next-generation sequencing, Scoring of sequence variants, Sub-Saharan Africa, South Africa, Nigeria, Sequence variants

## Abstract

**Background:**

The prevalence of Parkinson’s disease (PD) is increasing in sub-Saharan Africa, but little is known about the genetics of PD in these populations. Due to their unique ancestry and diversity, sub-Saharan African populations have the potential to reveal novel insights into the pathobiology of PD. In this study, we aimed to characterise the genetic variation in known and novel PD genes in a group of Black South African and Nigerian patients.

**Methods:**

We recruited 33 Black South African and 14 Nigerian PD patients, and screened them for sequence variants in 751 genes using an Ion AmpliSeq™ Neurological Research panel. We used bcftools to filter variants and *annovar* software for the annotation. Rare variants were prioritised using MetaLR and MetaSVM prediction scores. The effect of a variant on ATP13A2’s protein structure was investigated by molecular modelling.

**Results:**

We identified 14,655 rare variants with a minor allele frequency ≤ 0.01, which included 2448 missense variants. Notably, no common pathogenic mutations were identified in these patients. Also, none of the known PD-associated mutations were found highlighting the need for more studies in African populations. Altogether, 54 rare variants in 42 genes were considered deleterious and were prioritized, based on MetaLR and MetaSVM scores, for follow-up studies. Protein modelling showed that the *S1004R* variant in ATP13A2 possibly alters the conformation of the protein.

**Conclusions:**

We identified several rare variants predicted to be deleterious in sub-Saharan Africa PD patients; however, further studies are required to determine the biological effects of these variants and their possible role in PD. Studies such as these are important to elucidate the genetic aetiology of this disorder in patients of African ancestry.

## Background

Parkinson’s disease (PD) is a debilitating neurodegenerative disorder that impairs patients’ motor skills, and speech coordination. It is one of the leading causes of disability and mortality among neurological disorders globally [1]. The neuropathological hallmark of PD is the progressive loss of predominantly dopaminergic neurons of the *substantia nigra pars compacta* of the midbrain, which regulate voluntary movement. The diagnosis of this disorder is largely clinical using criteria such as the UK PD Society Brain Bank criteria (UKPDSBBC) to differentiate ageing related symptoms from PD [2]. The pathobiology is yet to be fully elucidated, but environmental and genetic factors have been linked to PD aetiology [3, 4]. PD symptoms usually manifest in the same way in all patients, but the prevalence, incidence and risk factors may vary according to the geographical region [5]. Estimates of PD prevalence in sub-Saharan Africa (SSA) vary widely across previous studies and range from 10 to 235/100,000 in urban populations [6, 7].

Genetics as an etiologic concept in PD has been well-established [8, 9]. Approximately 5–10% of PD patients have a familial form of the disease, which is due to highly penetrant, rare pathogenic mutations [9]. For sporadic forms of this disorder, the genetics is complex as common genetic variants may act in concert with environmental factors [9–11]. The genetic discoveries have led to important hypotheses about the mechanisms underlying PD, which include dysfunction of the ubiquitin–proteasome system and mitochondrial dysfunction coupled with oxidative stress [12].

Most of the studies on the established PD genes or genes associated with PD including *SNCA, LRRK2, PRKN, PINK1, PARK7, ATP13A2* and *GBA*, have been performed in European, North American, North African Arab or Asian populations [9, 13, 14]. In general, limited studies exist on the genetics of PD in the Black African populations [15]. It has been suggested that the variants most commonly associated with PD are rare among South African PD patients [16, 17]. Similarly, a previous genetic study screened for mutations in *LRRK2*, *PRKN* and *ATXN3* in 57 Nigerian PD patients but did not identify any pathogenic mutations [18]. African populations have a diverse ancestry, and have more private alleles than any other population, suggesting that the genetic aetiology of PD in African populations could be unique [19].

Next-Generation Sequencing (NGS) provides a way to explore the genetic basis of diseases, and has resulted in the discovery of a large number of disease-associated mutations [20]. In contrast to whole-genome or whole-exome sequencing [21, 22], targeted sequencing panels [23] focus the analysis on specific genes of interest. The Ion AmpliSeq™ Neurological Research Panel is a commercially available panel designed to screen genes linked to neurological disorders as well as genes involved in brain function. The primary goals of the present study were to use this panel to determine whether a common pathogenic mutation was present, and to characterise the genetic variation in known and novel PD genes, in a group of Black South African and Nigerian PD patients.

## Methods

### Study participants

The study group consisted of 33 unrelated Black South African PD patients and 14 unrelated Nigerian PD patients. South African patients were primarily recruited at the Neurology Clinic of Tygerberg Academic Hospital, Cape Town, South Africa, and at the Neurology Clinic of Frere Hospital, East London, South Africa. Nigerian PD patients were recruited at the Neurology Clinic, Obafemi Awolowo University Teaching Hospitals Complex, Ile-Ife, Nigeria. All patients were confirmed to have PD by neurologists, based on the UKPDSBBC diagnostic criteria. All patients provided written informed consent to take part in the study and provided peripheral blood samples for genetic studies. This study was approved by the Health Research Ethics Committee of Stellenbosch University (HREC 2002/C059, N16/04/041 and S16/08/151), and the Ethics and Research Committee of Obafemi Awolowo University Teaching Hospitals (ERC/2015/08/15). Demographic information and clinical characteristics of the patients are provided in Table 1 and Additional file 1: Table S1.

**Table 1:** Characteristics of the 47 Parkinson’s disease patients

Characteristic	Black South African *N* = 33	Nigerian *N* = 14
Sex, male, n (%)	18 (54)	11 (78)
Average age-at-onset ± SD (range), years	48 ± 8 (30–59)	63 ± 13 (36–80)
Average age at recruitment ± SD (range), years	55 ± 11 (35–78)	67 ± 11 (42–81)
Positive family history of PD, n (%)	2^a^ (6)	0

^a^ Individuals s43_059 and s94_069 have a possible Mendelian inheritance pattern for PD

### Quality control and annotation of targeted next-generation sequencing (tNGS) data

The Ion AmpliSeq™ Neurological Research panel and the Ion AmpliSeq™ Library Kit 2.0 (Thermo Scientific, Waltham, Massachusetts, USA) were used for multiplex PCR amplification of 751 genes (Additional file 2: Table S2). The intronic regions incorporated as part of the exon targets are listed separately in Additional file 3: Table S3. Details on the library construction and next-generation-sequencing protocols are available in Additional file 4.

The flow space calibration, base calling, alignment with the reference genome (GRCh38–hg19), coverage analysis and variant calling were performed using standard parameters in the Ion Torrent Software Suite (ISS) version 5.4.0. Sequenced variants, including insertions and deletions (INDELs), splice site variants, single nucleotide variants (SNVs), multiple nucleotide variants (MNVs), as well as variants in the 3′ untranslated region (UTR3) and 5′ untranslated region (UTR5) were identified. The variant call format (VCF) files produced by the ISS were filtered using *bcftools* to ensure that:

- FILTER = PASS (the ISS internal criteria for setting the PASS filter were met).
- QUAL > 100 (the quality score exceeded 100).
- FMT/AO ≥ 20 (there were at least 20 reads for the alternative allele).
- FMT/DP ≥ 40 (there were at least 40 reads in total).
- FMT/SAF ≥ 5 (there were at least 5 reads of the alternative allele in the forward direction).
- FMT/SAR ≥ 5 (there were at least 5 reads of the alternative allele in the reverse direction).

These criteria ensured that the observation was made in both directions with at least five reads in either direction, the overall depth was at least 40 and the alternative allele depth was at least 20.

The sequencing data on the 47 samples were of good overall quality. We plotted a graph for the target region coverage using the *bam files* generated by the Ion Torrent Variant Caller (Additional file 5: Figure S1). This graph showed that 41 samples had at least 80% coverage of the target region at an average read depth of 40X, three samples had a coverage of 78–79%, and another three samples had a coverage of 61–76%.

The VCF files were merged into a single file and processed with the utility *annovar* (*annovar.openbioinformatics.org**/)* to produce an annotation file for all the variants that passed the criteria above. All available annotations were included. These included conservation scores, allele frequencies and functional predictions (Additional file 6: Table S4). Perl (*https://www.perl.org/**)* was used to extract data and analyses were carried out in R (R Core Team, 2018) [24]. Variants were extracted as homozygous or heterozygous for the alternative allele. The quality scores for all the variants passing the filters were extracted and analysed in R. Using the *bam* files, *bedtools* was used to generate statistics on the coverage (depth of sequencing) for the regions in the *Ampliseq* capture panel as defined by the manufacturer’s bed file. We focused on variants that are rare in control populations as defined by a minor allele frequency (MAF) threshold of 0.01. We created global classifications of variants and generated a summary of variant types, to encode variants as synonymous, missense or frameshift in the variable amino acid class, insertion, deletion or substitution in the variable mutation type, as well as single or multiple base variants in the variable mutated base*.* We merged the variant summary (whether the variant was observed as a *homo*zygous or *hetero*zygous), with the annotation.

### Variant prioritisation

We used the prediction scores MetaLR and MetaSVM for selecting deleterious sequence variants (Additional file 6: Table S4). MetaLR and MetaSVM are themselves *ensembles* (composite models) of many other scores [25]. Currently, these two have the best performance on curated data sets (training and test) of non-synonymous variants that contain both deleterious (protein-function altering) and benign variants. We therefore used these to prioritize the rare variants in our data. Both metrics were scaled as probabilities [0, 1] with scores close to 1 indicating certainty that the variant is deleterious. We used a score of > 0.8 as a cut-off for including the variant into our list of rare “pathogenic variants” as recommended by Liu et al [25]. We also used 24 other variant scoring algorithms. We plotted the correlation matrix of all 26 scoring algorithm outputs used in our study (Additional file 7: Figure S2). We generated Radar plots (http://www.cmap.polytechnique.fr/~lepennec/R/Radar/RadarAndParallelPlots.html) for each variant likely to be deleterious to demonstrate the correlation among 17 different scoring algorithms. All scores were standardized to 0–1 scale with score 1 (furthest from the centre of the graph) indicating strongest evidence that the variant is deleterious.

### Protein structures and modelling for functional prediction

To determine the consequences introduced by potential pathogenic variants on the protein structure, we selected a variant in *ATP13A2*, for this analysis. We extracted protein information from the Protein Data Bank (PDB) of the Research Collaboratory for Structural Bioinformatics (RCSB) (https://www.rcsb.org/) [26]. The structure of ATP13A2 was modelled by submitting the 1180 amino acid UniProt accession ACQ9NQ11 to the Phyre2 server [27]. Phyre2 is a suite of tools available on the web to predict and analyse protein structure, function and mutations. The predicted ATP13A2 structure conformed well with those of known P-type ATPase cation pumps [28–31]. This structure was used for additional modelling using the Maestro 11.4 suite of software (Schrödinger Inc., Cambridge, MA). The protein parameterization was carried out using the Maestro Preparation Wizard. The Ca^2+^ binding sites were identified based on the availability of coordinating glutamate, aspartate, asparagine and glutamine sidechains as found in the other P-type ATPase structures. The two Ca^2+^ ions were manually docked into the active sites and the structure's energy minimized. The S1004R mutation was generated using in silico mutagenesis with subsequent energy minimization. Based on these structures, the interaction network between the mutated site and Ca^2+^ was identified (Kenyon et al. unpublished results).

## Results

The mean age-at-onset (AAO) of PD in patients varied between the two study groups. It was 48 ± 8 years and 63 ± 13 years for the South African and Nigerian patients, respectively (Table 1). This may be because recruitment in South Africa was predominantly focussed on patients with earlier AAO (< 50 years). Two of the South African PD patients had a positive family history with both having an affected sibling and an affected parent.

### Identification of sequence variants

**Fig. 1:**
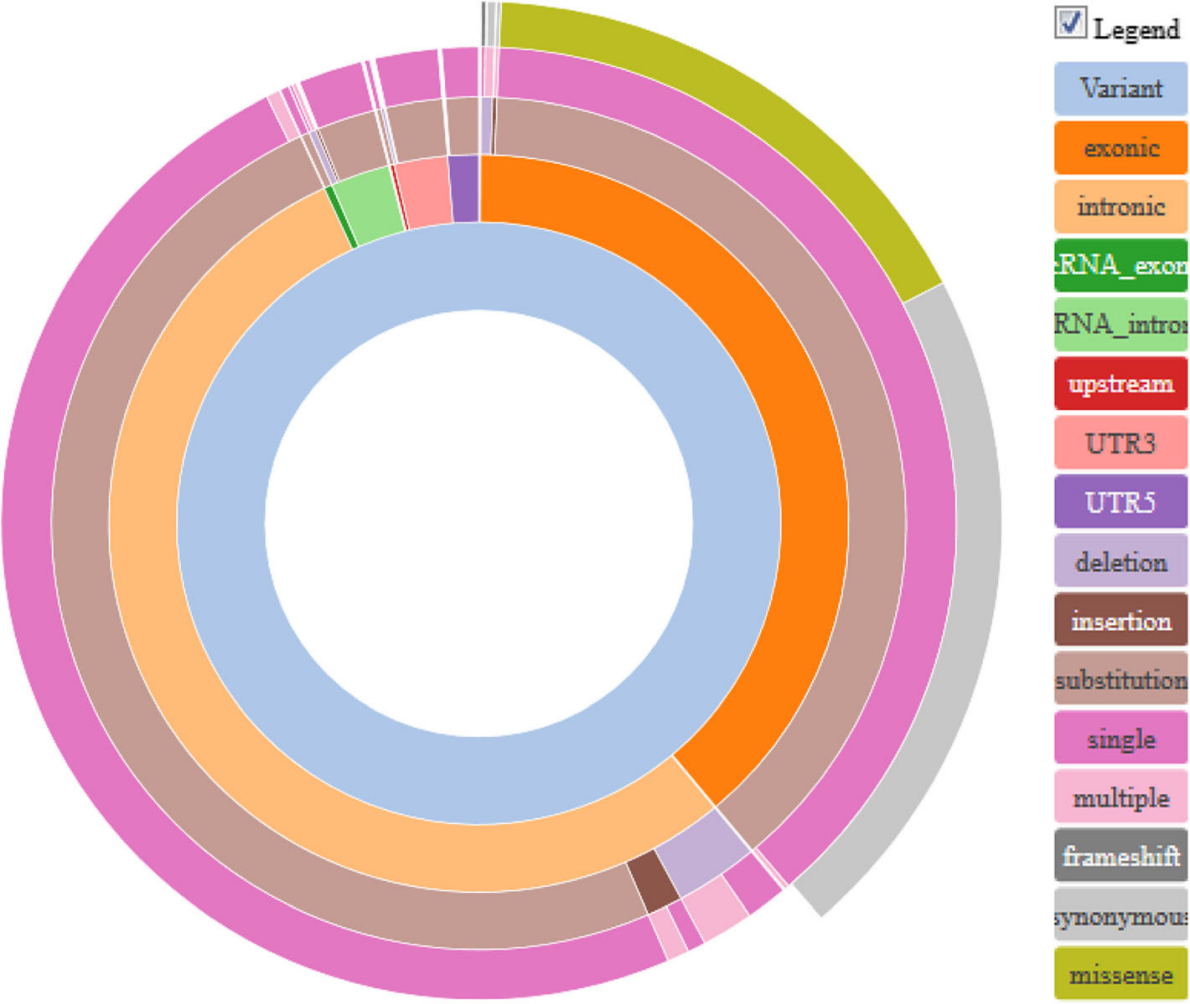
Sunburst diagram showing the functional classes of 14,655 rare (MAF ≤ 0 .01) sequence variants identified in 33 Black South African and 14 Nigerian PD patients. An interactive HTML-version of the figure is available at BMC website

We applied stringent threshold criteria for the filtering and annotation of the variants to exclude low quality variants. Altogether 25,917 sequence variants passed quality control. We then removed all variants with MAF > 0.01 in any of the sequencing databases used as reference databases for the study (Additional file 6: Table S4) and were left with 14,655 rare variants. These rare variants could be classified into 7934 intronic and 5695 exonic variants (Fig. 1; an interactive html-version of the figure is at BMC website). They included 198 UTR5 and 341 UTR3 variants, as well as 32 frameshift, 3175 synonymous and 2448 missense variants. Altogether 14,057 were SNVs and 598 MNVs. There were 261 insertions, 600 deletions and 13,794 substitutions.

In addition, we separately screened 16 PD genes on the panel (*SNCA, LRRK2, PRKN, PINK1, PARK7, ATP13A2, EIF4G1, GIGYF2, PLA2G6, FBXO7, VPS35, MAPT, HTRA2, SPG11, GRN* and *DCTN1*) for all sequence variants, and these results are shown in Additional file 8: Table S5).

### Pathogenicity prediction of variants

To determine which rare variants are likely to be deleterious and could potentially contribute to the PD pathobiology in the study participants, we used MetaLR and MetaSVM. We focused on identifying rare (MAF ≤ 0.01) or novel (not seen in any of the databases listed in Additional file 6: Table S4) exonic variants predicted to be deleterious. The goal was to minimize the number of false positives by applying stringent filtering criteria. Altogether, 52 heterozygous, one hemizygous and one homozygous exonic rare (MAF ≤ 0.01) missense variants predicted to be deleterious were found in 42 genes (Tables 2 and 3). This included a heterozygous missense variant in one of the known PD genes, *ATP13A2* (*S1004R*) which was validated by Sanger sequencing (data not shown). Radar plots demonstrating pathogenicity scores for each of these 54 rare variants are shown in Additional file 9: Figure S3.

**Table 2:** List of 54 rare variants predicted to be deleterious using MetaLR and MetaSVM

Chr	Start	Gene Symbol	Ref allele	Alt allele	Variant	MetaLR score	MetaSVM score	CountNGR patients	CountSA patients	CountAll patients	GnomAD MAF (Controls, All)	GnomAD MAF (Controls, African)	rs number	Gene linked to PD
16	70,296,316	*AARS*	T	A	Y535F	0.906	0.836	1	0	1	9.14e-6	0.000	rs756650948	
22	40,755,001	*ADSL*	G	T	A206S	0.914	0.976	0	1	1	4.16e-4	0.005	rs148411623	
5	125,919,688	*ALDH7A1*	C	T	R110Q	0.980	0.966	0	1	1	3.98e-06	0.000	rs1160207513	
X	66,863,156	*AR*	A	T	T559S	0.918	0.858	0	1	1	1.14e-4	0.001	rs139756052	
22	51,063,778	*ARSA*	T	C	N442S	0.847	0.816	0	1	1	3.25e-4	0.003	rs6151427	
22	51,065,288	*ARSA*	G	T	P220T	0.983	0.969	1	0	1	5.82e-5	7.00e-4	rs146173768	
1	17,313,614	*ATP13A2*	T	G	S1004R	0.905	0.844	0	1	1	4.032e-06	0.000	rs1230099396	Yes
16	28,912,085	*ATP2A1*	G	A	D525N	0.800	0.832	0	1	1	1.65e-3	0.018	rs74573581	
11	62,458,888	*BSCL2*	A	G	L290P	0.880	0.818	1	0	1	N/A	N/A	rs1451786763	
15	42,693,950	*CAPN3*	G	A	R441Q	0.874	0.811	1	1	2	3.33e-5	2.05e-4	rs147764579	
10	50,828,566	*CHAT*	T	G	M84R	0.892	0.828	0	1	1	9.15e-5	0.001	rs376808313	
1	154,544,030	*CHRNB2*	C	T	P244L	0.944	0.988	1	0	1	9.14e-6	0.000	rs1462718185	
15	68,500,645	*CLN6*	T	C	S257G	0.814	0.811	1	1	2	0.001	0.014	rs151295143	
21	47,544,826	*COL6A2*	G	A	G588S	1.000	0.987	0	1	1	8.23e-5	8.00e-4	rs139488626	
2	238,258,801	*COL6A3*	G	A	R1683C	0.975	0.933	0	1	1	9.98e-5	3.00e-4	rs116608946	
3	148,899,821	*CP*	T	C	E842G	0.931	0.934	1	0	1	0.001	0.011	rs149858116	
19	10,893,647	*DNM2*	G	T	V234 L	0.933	0.860	0	1	1	4.57e-5	0.000	rs377159042	
6	56,765,318	*DST*	A	C	S106R	0.825	0.858	1	0	1	5.86e-5	7.00e-4	rs375833647	Yes
6	56,765,371	*DST*	C	T	A89T	0.805	0.824	0	1	1	3.35e-4	3.00e-4	rs370358616	
X	153,583,294	*FLNA*	C	T	G1698S	0.910	0.858	0	1	1	7.52e-5	0.000	rs781993685	Yes
X	153,592,919	*FLNA*	G	A	A666V	0.872	0.829	0	1	1	1.00e-4	1.76e-4	rs374295965	
14	88,414,158	*GALC*	G	C	T445S	0.824	0.837	0	3	3	0.001	0.016	rs34134328	Yes
9	36,236,974	*GNE*	A	C	D203E	0.858	0.820	0	3	3	0.001	0.013	rs35224402	
17	10,443,936	*MYH2*	T	C	D328G	0.982	0.935	0	1	1	N/A	N/A	N/A	
17	10,314,218	*MYH8*	A	C	L488R	0.967	0.881	0	1	1	N/A	N/A	N/A	
12	4,763,994	*NDUFA9*	G	A	R75H	0.901	0.879	1	0	1	8.97e-4	0.008	rs35263902	
20	13,797,783	*NDUFAF5*	G	C	G294A	0.981	0.919	0	2	2	4.57e-5	6.98e-4	rs140825882	Yes
1	161,182,208	*NDUFS2*	C	G	P352A	0.928	0.865	0	1	1	0.073	0.016	rs11576415	
11	66,618,540	*PC*	G	C	R732G	0.878	0.882	0	2	2	9.21e-6	0.000	rs112948607	
15	89,868,793	*POLG*	G	A	H613Y	0.854	0.858	1	0	1	6.26e-4	0.006	rs147407423	Yes
3	64,133,345	*PRICKLE2*	T	G	Q274P	0.904	0.838	0	1	1	N/A	N/A	rs564701683	
14	73,659,375	*PSEN1*	T	C	V191A	0.992	0.953	0	2	2	N/A	N/A	rs112451138	
1	227,073,297	*PSEN2*	G	A	V139 M	0.963	0.948	0	1	1	1.46e-4	0.000	rs202178897	
1	227,079,048	*PSEN2*	A	G	Y319C	0.950	0.914	0	1	1	2.78e-5	4.34e-4	rs547494670	
19	12,921,137	*RNASEH2A*	C	T	R186W	0.957	0.910	0	1	1	4e-06	0.000	rs77103971	
19	38,974,116	*RYR1*	C	T	P1632S	0.858	0.908	1	2	3	1.67e-3	0.020	rs76537615	
19	38,998,362	*RYR1*	G	A	D2943N	0.904	0.895	0	2	2	8.31e-4	0.010	rs79294840	
19	39,019,242	*RYR1*	C	G	H3642Q	0.873	0.853	0	2	2	9.15e-4	0.011	rs114351116	
19	39,025,421	*RYR1*	C	T	A3769V	0.837	0.846	0	1	1	1.42e-4	0.001	rs146361173	
20	35,533,822	*SAMHD1*	T	G	N452 T	0.828	0.822	0	1	1	N/A	N/A	N/A	
17	62,019,123	*SCN4A*	C	T	V1507I	0.880	0.843	0	1	1	5.82e-5	6.16e-4	rs140517911	
17	62,036,686	*SCN4A*	C	A	S653I	0.982	0.976	0	1	1	N/A	N/A	rs535473662	
17	62,049,961	*SCN4A*	C	G	E81Q	0.853	0.912	2	2	4	1.34e-3	0.016	rs111926172	
4	52,895,854	*SGCB*	T	C	N140S	0.847	0.872	0	1	1	4.16e-5	0	rs775409967	
15	34,534,333	*SLC12A6*	C	A	G696 V	0.957	0.934	0	1	1	N/A	N/A	rs369367800	
15	34,542,869	*SLC12A6*	A	C	I503M	0.859	0.910	0	1	1	N/A	N/A	N/A	
6	152,809,602	*SYNE1*	A	G	W333R	0.925	0.865	0	1	1	1.34e-4	0.002	rs146668256	
20	2,376,062	*TGM6*	T	A	L135H	0.984	0.957	0	1	1	9.15e-5	0.001	rs138009191	
11	61,160,781	*TMEM216*	A	G	E38G	0.848	0.813	1	0	1	1.25e-4	0.001	rs568253718	
8	94,827,551	*TMEM67*	G	A	S928 N	0.822	0.838	0	1	1	3.05e-5	4.45e-4	rs538380011	
16	2,130,346	*TSC2*	G	T	G949 V	0.952	0.895	0	1	1	1.42e-4	3.59e-4	rs137854262	
16	2,134,230	*TSC2*	C	T	S1092 L	0.871	0.819	0	1	1	1.71e-4	0.002	rs148527903	
16	2,138,318	*TSC2*	C	T	R1507C	0.936	0.889	1	0	1	1.83e-5	0.000	rs781630603	
2	179,590,564	*TTN*	C	T	G5585S	0.904	0.823	0	1	1	2.77e-5	2.80e-4	rs139549363	

*Chr* Chromosome, *Start* GRCh38 coordinates, *Ref allele* Reference allele based on GRCh38, *Alt allele* Alternative (variant) allele found in this study, *CountNGR* Number of variant alleles in the Nigerian sample, *CountSA* Number of variant alleles in the South African sample, *CountAll* Number of variant alleles in the entire study sample, *GnomAD MAF (Controls, All)* Minor allele frequency of healthy controls from all population groups (*n* = 60,146 controls) on the public database GnomAD (https://gnomad.broadinstitute.org/), *GnomAD MAF (Controls, African)* Minor allele frequency of healthy controls from the African/African American subset (*n* = 8128 controls) on the public database GnomAD, *N/A* Not available

**Table 3:**
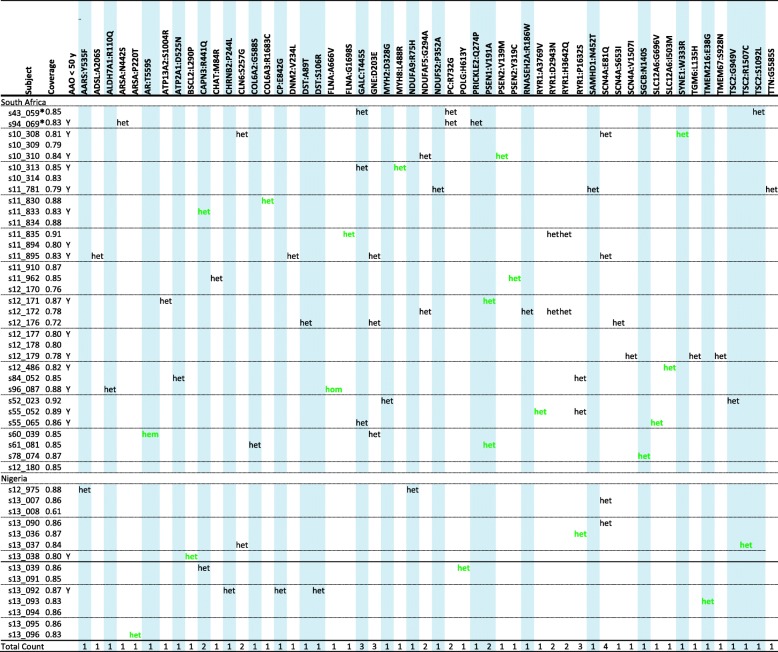
Rare deleterious variants identified in the study participants

Subject, sample code; Coverage, global tNGS data coverage for the listed sample; AAO < 50 y, cases with early-onset PD are indicated (Y, yes); Other column headers indicate gene and variant for which data are provided. Hem, patient was hemizygous for the variant; het, patient was heterozygous for the variant; hom, patient was homozygous for the variant. TOTAL COUNT, number of each rare deleterious variant in the study population. *, These individuals have a positive family history of PD. The variants in bold and green font are the candidate variants prioritized for further analyses

We then analysed separately the two South African individuals (S43_059 and S94_069) with a positive family history of PD (one affected sibling and an affected parent). They each had three heterozygous variants (Table 3). Both individuals had a pyruvate carboxylase (*PC*) R732G variant (rs112948607). Additionally, individual S43_059 carried galactosylceramidase (*GALC*) T445S (rs34134328) and TSC complex subunit 2 (*TSC2*) S1092 L (rs148527903) variants; while S94_069 carried arylsulfatase A (*ARSA*) N442S (rs6151427) and prickle planar cell polarity protein 2 (*PRICKLE2*) Q274P (rs564701683) variants. All of the variants had a high certainty of being predicted to be deleterious (pathogenicity score > 0.8) (Table 2). The *GALC* variant was excluded based on its high MAF in GnomAD African controls (MAF = 0.016) but all of the other variants are rare (MAF < 0.01) ([32]; Table 2) and are therefore potential candidates. Notably, the *PC* R732G variant that they both share was not found in any of the other patients screened. However, since there was only DNA available for one affected sibling for each of these patients, co-segregation analysis of the variants with disease could not be performed.

We attempted to prioritise one possible pathogenic variant per patient based on MAF (< 0.01), pathogenicity prediction scores (> 0.8) and evidence of prior association of the gene/protein with PD or Parkinsonism (Table 2; Additional file 10: Table S6). In some cases, the MAF of the variant in African controls in GnomAD was ≥0.01, similar to the frequency observed in the patients (Table 2), and those variants were therefore excluded. The prioritised variants are shown in bold and in green font in Table 3. In a few individuals, one variant could not be prioritised over others as more than one variant fulfilled these criteria.

### Protein modelling for the S1004R variant in *ATP13A2*

When the S1004R variant was inserted into the ATP13A2 structure and energy minimized (Kenyon et al. unpublished results), we found that the peptide backbone around the cation binding site was displaced, changing the distance between the cation and a coordinating atom from 3.44 to 2.66 Å (Additional file 11: Figure S4). It could be postulated that the conformational change may alter the efficiency of the pump by interfering with the reaction cycle [28–30].

## Discussion

In this study, 47 Black South African and Nigerian PD patients were screened and 54 potentially deleterious sequence variants with MAF ≤ 0.01 in 42 different genes were identified. The 751-gene panel used in the current study contains only 16 of the 34 known PD genes, but it does have six genes (*ATP13A2*, *LRRK2*, *PARK7, PINK1*, *PRKN*, and *SNCA)* with strong prior evidence of being involved in PD pathobiology. We identified a rare sequence variant predicted to be deleterious in only one of these genes, *ATP13A2*. Notably, we did not identify any of the previously reported pathogenic PD mutations catalogued in the PDmutDB database (https://www.molgen.vib-ua.be/PDMutDB/database) in the SSA patients. One possible reason is that, as seen in previous genetic studies on SSA PD patients, common mutations such as *LRRK2* G2019S may be a rare cause of PD in these populations [15].

Protein modelling analysis of the *ATP13A2* S1004R variant, which was found in a South African patient (AAO of 39 years), revealed that the variant is potentially functionally important. An interaction between R1004 and the cation binding site was identified suggesting that the variant would interfere with the function of ATP13A2 as a pump of inorganic cations such as metal ions. A previous functional study demonstrated that increased expression of ATP13A2 supresses α-synuclein toxicity in neural cells and that ATP13A2 was likely to act as a Zn^2+^ pump [33]. Thus, this variant could potentially contribute to PD however, wet-laboratory functional studies are necessary to prove that the variant is indeed pathogenic.

In the two patients with a possible Mendelian inheritance of PD, five heterozygous variants were identified. Co-segregation analysis of the variants with disease in these families was not possible due to a lack of DNA of the family members. However, none of the genes in which these variants were found has been linked to Mendelian forms of PD. In fact, mutations within the *PC*, *PRICKLE2* and *TSC2* genes have previously been associated with non-neurodegenerative diseases including diseases involved in energy deficiency, tumour formation and seizures [34–36]. Therefore, it is unlikely that mutations in these genes would contribute to a Mendelian inheritance pattern of PD in these patients. *ARSA* mutations, similar to *GBA* mutations, have been previously linked to lysosomal storage diseases (LSDs) [37]. Although lysosomal mechanisms are increasingly being shown to be important in PD pathogenesis, the interplay between genetic mutations, lysosomal storage biology and PD is complex and require further elucidation to understand the underlying biology connecting lysosomal storage and PD. However, there is no evidence currently indicating that *ARSA* mutations cause familial forms of PD.

Limitations of our study include the fact that the sample size was small making it difficult to estimate the actual contribution of genetic factors to PD in the SSA populations. The belief among Black SSA populations that PD is caused by witchcraft and does not have a genetic link [38] and the notion that it is part of normal ageing, may have contributed to the difficulty in recruiting more patients for the study. Also, 18 of the 34 previously identified PD genes were not on the panel. In addition, annotation of sequence variants in terms of effect on the protein using bioinformatic tools remains problematic. We chose to use the MetaLR and MetaSVM algorithms that currently appear to perform best, but functional studies are needed for validation of these results. Future studies will involve using a custom-panel that captures all of the known PD genes; using whole-exome or whole-genome sequencing; screening of the patients for copy number variations especially in the *PINK1* and *PRKN* genes; and recruitment of a large number of ethnic-matched controls to determine the frequency of prioritized variants in these populations.

## Conclusions

Studies are urgently needed to characterise the genetic variation in the known as well as novel PD genes in the understudied SSA populations. Multi-national collaborations across Africa are essential to recruit the large numbers of patients and controls required. The current study provides a starting point to address this need and although it is acknowledged that the sample sizes used here are relatively small, the use of NGS technologies means that the full spectrum of sequence variation in 751 genes has been captured and is available for future studies. We identified several rare variants predicted to be deleterious and they provide new putative candidates for PD but further studies are required to assess their role in PD pathobiology. It is important to include SSA populations in PD genetic studies to ensure that they do not miss out on the potential benefits and opportunities promised by precision medicine [39].

## Supplementary information

**Additional file 1: Table S1.** Individual clinical data on study participants.

**Additional file 2: Table S2.** Neurological Research panel information provided by Ion AmpliSeq™ (www.ampliseq.com).

**Additional file 3: Table S3.** Intron regions covered as part of the exon targets.

**Additional file 4.** Protocols for library construction and Ion Torrent sequencing.

**Additional file 5: Figure S1.** Target region coverage for the 47 samples.

**Additional file 6: Table S4.** Tools and databases used for the annotation of sequence variants.

**Additional file 7: Figure S2.** Correlation between prediction and conservation scores.

**Additional file 8: Table S5.** Sequence variants found in the known PD genes.

**Additional file 9: Figure S3.** Radar plots for 54 selected rare variants.

**Additional file 10: Table S6.** Candidate genes with a link to Parkinson’s disease or Parkinsonism.

**Additional file 11: Figure S4.** Stereoscopic crystal structure models of ATP13A2 showing the position of S1004R.

## Data Availability

The *bam* files of the tNGS data have been deposited to the European Nucleotide Archive (ENA) and can be retrieved with an accession number PRJEB30330 from https://www.ebi.ac.uk/ena/browse/data-retrieval-rest.

## Abbreviations

AAO: Age at onset
ATP13A2: ATPase cation transporting 13A2
ATXN3: Ataxin 3
BLAST: Basic local alignment search tool
GBA: Beta-glucocerebrosidase
HMM: Hidden markov model
INDEL: Insertion and deletion
ISS: Ion Torrent software suite
LRRK2: Leucine-rich repeat kinase 2
MAF: Minor allele frequency
MNV: Multiple nucleotide variant
NGS: Next-generation sequencing
PARK7: Parkinsonism associated deglycase
PD: Parkinson’s disease
PDmutDB: Parkinson’s disease mutation database
PINK1: PTEN induced putative kinase 1
PRKN: Parkin RBR E3 ubiquitin protein ligase
SNCA: Alpha-synuclein
SNV: Single nucleotide variant
SSA: Sub-Saharan Africa
tNGS: Targeted NGS
UKPDSBBC: UK PD society brain bank criteria
UTR3: 3′ untranslated region
UTR5: 5′ untranslated region
VCF: Variant call format
