# Supplementary material for: Targeted next-generation sequencing identifies novel variants in candidate genes for Parkinson’s disease in Black South African and Nigerian patients
Source: BMC Med Genet. 2020 Feb 4;21:23. doi: 10.1186/s12881-020-0953-1 (PMC7001245; doi:10.1186/s12881-020-0953-1)
Supplement: Supplementary file 1 — Additional file 1: Table S1. Individual clinical data on study participants. [file 12881_2020_953_MOESM1_ESM.pdf]

Table S1. Individual clinical data on study participants

Sample code	Sex	Ethnicity/Country	AAO (y)	AAD (y)	Family history of PD
s43_59	M	Black/SA	51	61	Yes
s52_23	M	Black/SA	50	57	
s55_52	M	Black/SA	42	49	
s55_65	M	Black/SA	40	42	
s60_39	M	Black/SA	55	56	
s61_81	M	Black/SA	55	57	
s78_74	F	Black/SA	56	63	
s84_52	F	Black/SA	57	61	
s94_69	F	Black/SA	30	36	Yes
s96_87	M	Black/SA	37	38	
s10_308	F	Black/SA	49	77	
s10_309	M	Black/SA	52	59	
s10_310	M	Black/SA	45	48	
s10_313	F	Black/SA	42	49	
s10_314	F	Black/SA	55	57	
s11_781	F	Black/SA	31	35	
s11_830	F	Black/SA	57	60	
s11_833	M	Black/SA	48	50	
s11_834	M	Black/SA	52	56	
s11_835	F	Black/SA	52	60	
s11_894	M	Black/SA	44	49	
s11_895	M	Black/SA	49	53	
s11_910	F	Black/SA	57	62	
s11_962	M	Black/SA	53	77	
s12_170	F	Black/SA	52	62	
s12_171	M	Black/SA	39	78	
s12_172	F	Black/SA	55	56	
s12_176	F	Black/SA	51	55	
s12_177	M	Black/SA	45	55	
s12_178	F	Black/SA	53	55	
s12_179	F	Black/SA	30	35	
s12_180	M	Black/SA	55	55	
s12_486	M	Black/SA	35	49	
s12_975	F	Yoruba/N	80	80	
s13_007	M	Yoruba/N	52	59	
s13_008	M	Yoruba/N	62	66	
s13_036	M	Yoruba/N	57	64	
s13_037	M	Yoruba/N	79	81	
s13_038	M	Yoruba/N	36	42	
s13_039	F	Yoruba/N	71	73	
s13_090	M	Yoruba/N	59	60	
s13_091	M	Yoruba/N	79	80	
s13_092	M	Yoruba/N	48	59	
s13_093	M	Yoruba/N	53	64	
s13_094	F	Yoruba/N	62	64	
s13_095	M	Yoruba/N	73	81	
s13_096	M	Yoruba/N	68	71	

AAO, age at onset; AAD, age at diagnoses; F, female; M, male; N, Nigerian; SA, South African.
