# Supplementary material for: Targeted next-generation sequencing identifies novel variants in candidate genes for Parkinson’s disease in Black South African and Nigerian patients
Source: BMC Med Genet. 2020 Feb 4;21:23. doi: 10.1186/s12881-020-0953-1 (PMC7001245; doi:10.1186/s12881-020-0953-1)
Supplement: Supplementary file 4 — Additional file 4. Protocols for library construction and Ion Torrent sequencing. [file 12881_2020_953_MOESM4_ESM.pdf]

Supplementary Methods:

Protocols for library construction and Ion Torrent sequencing

Blood samples of study participants were collected at the recruitment sites into ethylenediaminetetraacetic acid (EDTA) tubes and genomic DNA (gDNA) were extracted using the Nucleo Spin Blood XL kit (Macherey-Nagel Duren, Germany). gDNA was quantified with a Qubit 2.0 Fluorometer using the Qubit dsDNA HS assay kit according to the manufacturer's protocol, MAN0002326 REVA.0. Quality scores were determined on the LabChip GXII Touch using the DNA Extended Range LabChip and the gDNA Reagent Kit according to the protocol CLS140166, Rev. C (PerkinElmer, USA).
The Ion AmpliSeq™ Neurological Research panel and the Ion AmpliSeq™ Library Kit 2.0 (Thermo Scientific, Waltham, Massachusetts, USA) were used for multiplex PCR amplification of 751 genes (see Additional file 2: Table S2; https://www.ampliseq.com/tmpl/view.action?tmplDesignId=62969292#/?listAction=tmplCoverageSummaryList&tmplDesignId=62969292&wrapperId=ajaxTableWrapper) and library construction prior to sequencing on the Ion S5™.

In addition to targeting all coding regions, an additional 101 non–coding disease–causing loci were targeted, as well as 14 repeat-expansion regions in genes *AFF2*, *AR*, *ATXN1*, *ATXN7*, *ATXN8OS*, *ATXN3*, *CACNA1A*, *CSTB*, *DMPK*, *HTT*, *JPH3*, *PABPN1*, *PPP2R2B*, and *TBP*. Among the 751 neurological genes on the panel, there are 16 genes which have been previously reported to contain mutations in PD patients. These genes are: *SNCA*, *LRRK2*, *PRKN, PINK1, PARK7, ATP13A2, EIF4G1, GIGYF2, PLA2G6, FBXO7, VPS35, MAPT, HTRA2, SPG11, GRN* and *DCTN1*.

Using two primer pools with a 20 ng input gDNA template, the target regions were amplified in 13 cycles, consisting of 15 s at 99°C and 16 min at 60°C, on the SimpliAmp Thermal Cycler Kit (Thermo Fisher Scientific, Waltham, MA, USA) using the Ion AmpliSeq™ Neurological Research Panel according to the manufacturer's protocol, MAN0006735, REVE.0 (www.ampliseq.com). Following amplification with the two primer pools, the products were combined and primer sequences partially digested by the IonShear™ Plus Enzyme Mix II. Barcode adapters were used to generate adapter-ligated libraries. The libraries were purified with Agencourt™ AMPure™ XP reagent and eluted in 50 µl low-Tris-EDTA buffer.

The AmpliSeq™ library was quantified using the Ion Library TaqMan™ Quantitation Kit (Thermo Fisher Scientific, Waltham, MA, USA) according to the manufacturer's protocol, MAN0015802 REVA.0. Quantitative PCR amplification was performed using the StepOnePlus™ Real–time PCR system (Thermo Scientific, Waltham, Massachusetts, USA). Libraries were diluted to 60 pM. The diluted, barcoded libraries were combined in equivamolar amounts using the Ion520™ and Ion530™ Chef Kit. In brief, 25 µl of pooled diluted library were loaded on the IonChef liquid handler using reagents, solutions and supplies according to the manufacturer's protocol, MAN0010846, REVD.0. Enriched, template-positive ion sphere particles were loaded onto two Ion530™ Chips. Massively parallel sequencing was performed on the Ion S5™ System using the Ion S5™ Sequencing Solutions and Sequencing Reagent Kits according to the manufacturer's protocol, MAN0010846 REV D.0 (Thermo Fisher Scientific, Waltham, Massachusetts, USA) at the DNA Sequencing Unit, Central Analytical Facilities of Stellenbosch University, Stellenbosch, South Africa.
