# Supplementary material for: Targeted next-generation sequencing identifies novel variants in candidate genes for Parkinson’s disease in Black South African and Nigerian patients
Source: BMC Med Genet. 2020 Feb 4;21:23. doi: 10.1186/s12881-020-0953-1 (PMC7001245; doi:10.1186/s12881-020-0953-1)
Supplement: Supplementary file 5 — Additional file 5: Figure S1. Target region coverage for the 47 samples. [file 12881_2020_953_MOESM5_ESM.pdf]

Target Region Coverage for Set 1

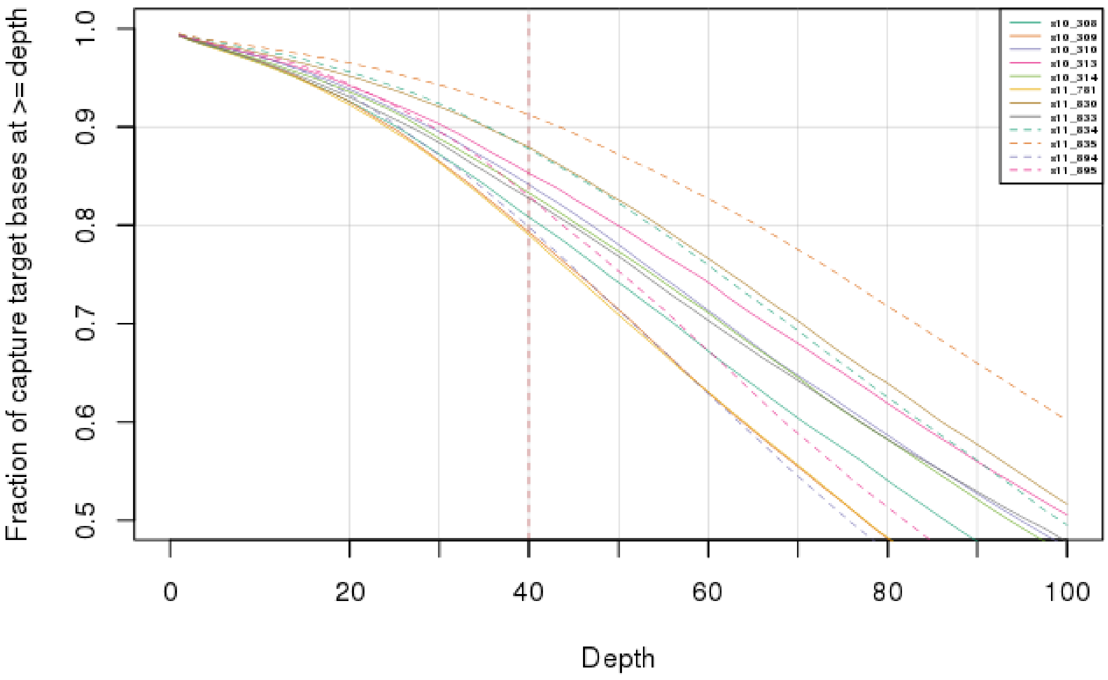

Target Region Coverage for Set 2

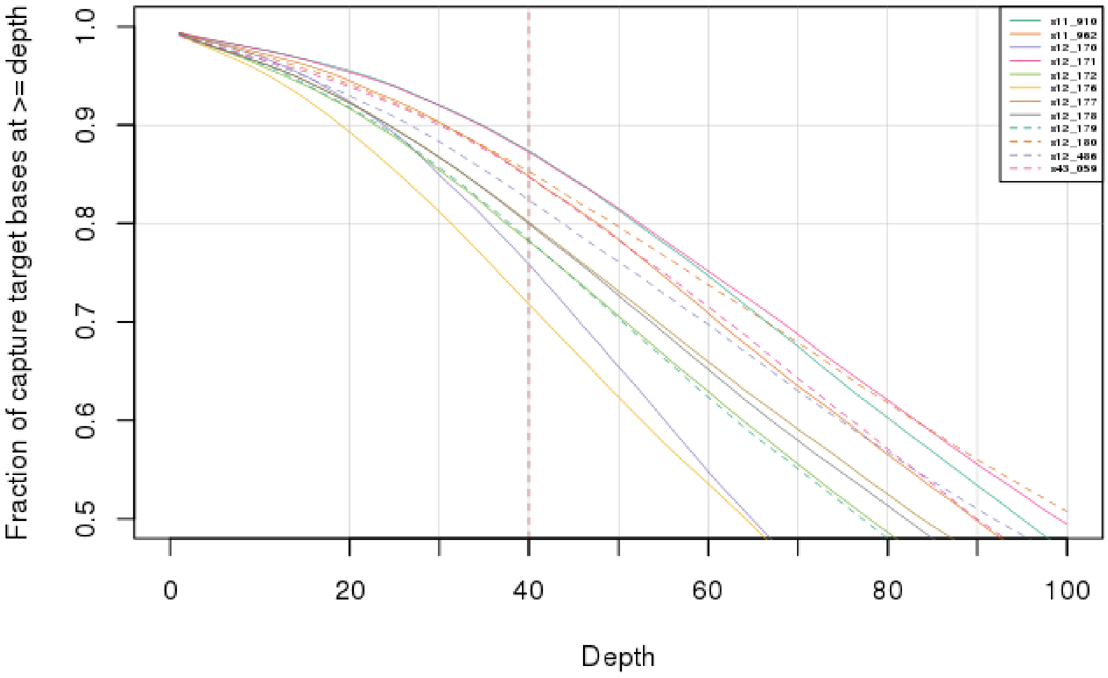

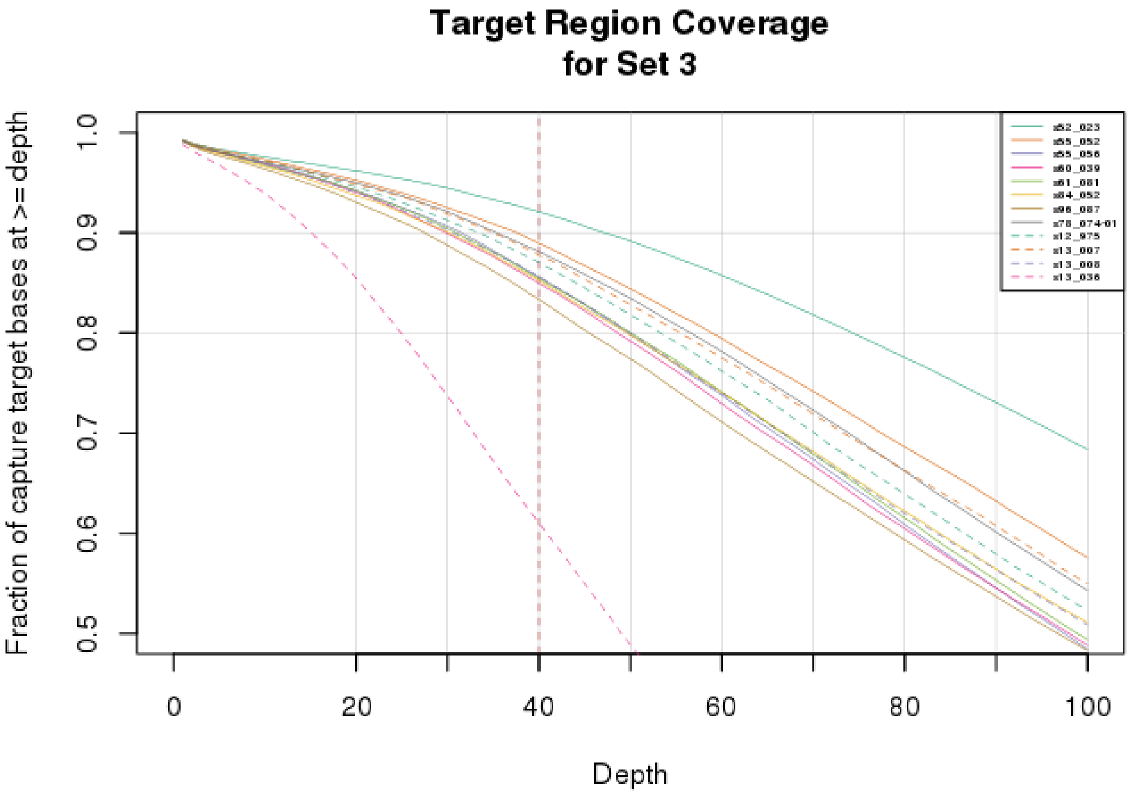

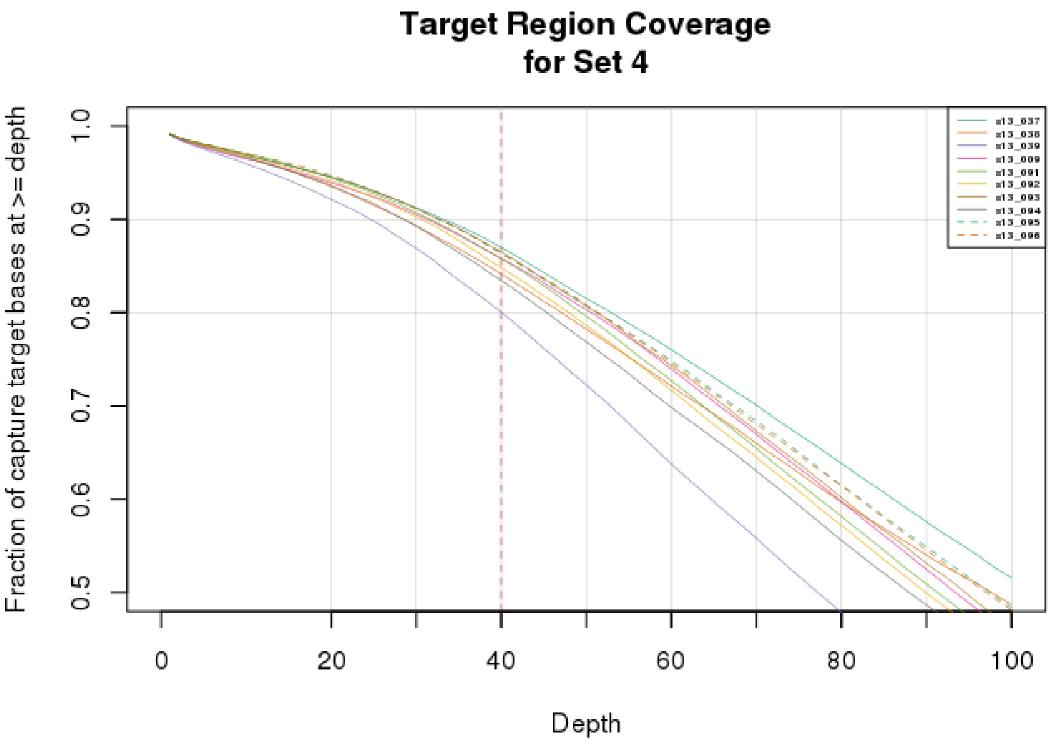

FIGURE S1. Target region coverage for the 47 samples. Sets 1-4 represent the samples binned into sets of up to12 samples each. Most (41/47) of the samples show coverage of 80% of the target region at a depth of 40x. Sample codes are shown in the insert.
