# Supplementary material for: Targeted next-generation sequencing identifies novel variants in candidate genes for Parkinson’s disease in Black South African and Nigerian patients
Source: BMC Med Genet. 2020 Feb 4;21:23. doi: 10.1186/s12881-020-0953-1 (PMC7001245; doi:10.1186/s12881-020-0953-1)
Supplement: Supplementary file 6 — Additional file 6: Table S4. Tools and databases used for the annotation of sequence variants. [file 12881_2020_953_MOESM6_ESM.pdf]

Table S4: Tools and databases used for the annotation of sequence variants

Annovar Name	Description	Source
**RefSeq Annotation**		
Gene.refGene	RefGene gene Symbol	https://www.ncbi.nlm.nih.gov/refseq
GeneDetail.refGene	Variant in HGNC Notation per transcript	https://www.ncbi.nlm.nih.gov/refseq
ExonicFunc.refGene	Type of mutation (synonymous, frameshift, etc.)	https://www.ncbi.nlm.nih.gov/refseq
AAChange.refGene	Variant at protein level in HGNC Notation	https://www.ncbi.nlm.nih.gov/refseq
cytoBand	Cytological Bands	http://www.software.broadinstitute.org/software/igv/cytoband
genomicSuperDups	Known Large Duplications	http://varianttools.sourceforge.net/Annotation/GenomicSuperDups
**SNP databases**		
snp142	SNP name in dbSNP v142	http://www.ncbi.nlm.nih.gov/snp
avsnp142	SNP name	http://www.ncbi.nlm.nih.gov/snp
**Frequencies**		
X1000g2015aug_all	1000 Genomes Frequencies All	http://www.internationalgenome.org/category/population
X1000g2015aug_afr	1000 Genomes Frequencies African	http://www.internationalgenome.org/category/population
X1000g2015aug_eas	1000 Genomes Frequencies East Asian	http://www.internationalgenome.org/category/population
X1000g2015aug_amr	1000 Genomes Frequencies American	http://www.internationalgenome.org/category/population
Kaviar_AF	Kaviar Frequencies	http://www.db.systemsbiology.net/kaviar
Kaviar_AC	Kaviar Variant Count	http://www.db.systemsbiology.net/kaviar
Kaviar_AN	Kaviar Total Allele Count	http://www.db.systemsbiology.net/kaviar
cg69	Cancer Genome Frequencies	http://www.completegenomics.com/public-data/69-genomes/
gnomAD_genome_ALL	Genome Aggregation Database (gnomAD) Genome Frequencies All	http://gnomad.broadinstitute.org/
gnomAD_genome_AFR	gnomAD Genome Frequencies African	http://gnomad.broadinstitute.org/
gnomAD_genome_AMR	gnomAD Genome Frequencies American	http://gnomad.broadinstitute.org/
gnomAD_genome_ASJ	gnomAD Genome Frequencies Ashkenazi Jewish	http://gnomad.broadinstitute.org/
gnomAD_genome_EAS	gnomAD Genome Frequencies East Asian	http://gnomad.broadinstitute.org/
gnomAD_genome_FIN	gnomAD Genome Frequencies Finnish	http://gnomad.broadinstitute.org/
gnomAD_genome_NFE	gnomAD Genome Frequencies Non-Finnish European	http://gnomad.broadinstitute.org/
gnomAD_genome_OTH	gnomAD Genome Frequencies Other	http://gnomad.broadinstitute.org/
esp6500siv2_all	esp 6500 Frequencies All	http://www.evs.gs.washington.edu
esp6500siv2_aa	esp 6500 Frequencies African American	http://www.evs.gs.washington.edu
esp6500siv2_ea	esp 6500 Frequencies European American	http://www.evs.gs.washington.edu
gnomAD_exome_ALL	gnomAD Exome Frequencies All (as above)	http://gnomad.broadinstitute.org/
gnomAD_exome_AFR	gnomAD Exome Frequencies	http://gnomad.broadinstitute.org/
gnomAD_exome_AMR	gnomAD Exome Frequencies	http://gnomad.broadinstitute.org/
gnomAD_exome_ASJ	gnomAD Exome Frequencies	http://gnomad.broadinstitute.org/

Annovar Name	Description	Source
gnomAD_exome_EAS	gnomAD Exome Frequencies	http://gnomad.broadinstitute.org/
gnomAD_exome_FIN	gnomAD Exome Frequencies	http://gnomad.broadinstitute.org//
gnomAD_exome_NFE	gnomAD Exome Frequencies	http://gnomad.broadinstitute.org/
gnomAD_exome_OTH	gnomAD Exome Frequencies	http://gnomad.broadinstitute.org/
gnomAD_exome_SAS	gnomAD Exome Frequencies South Asian	http://gnomad.broadinstitute.org/
ExAC_ALL	ExAC Frequencies All	http://exac.broadinstitute.org/
ExAC_AFR	ExAC Frequencies African	http://exac.broadinstitute.org/
ExAC_AMR	ExAC Frequencies American	http://exac.broadinstitute.org/
ExAC_EAS	ExAC Frequencies East Asian	http://exac.broadinstitute.org/
ExAC_FIN	ExAC Frequencies Finnish	http://exac.broadinstitute.org/
ExAC_NFE	ExAC Frequencies Non-Finnish European	http://exac.broadinstitute.org/
ExAC_OTH	ExAC Frequencies Other	http://exac.broadinstitute.org/
ExAC_SAS	ExAC Frequencies South Asian	http://exac.broadinstitute.org/
**Functional Annotation**		
cosmic70	Cosmic database	https://cancer.sanger.ac.uk/cosmic/
CLINSIG	Clinical significance in ClinVar	www.openbioinformatics.org/annovar
CLNDBN	Variant disease name	www.openbioinformatics.org/annovar
CLNACC	Variant Accession and Versions	www.openbioinformatics.org/annovar
CLNDSDB	Variant disease database name	www.openbioinformatics.org/annovar
CLNDSDBID	Variant disease database ID	www.openbioinformatics.org/annovar
ICGC_Id	International cancer genome consortium ID	www.openbioinformatics.org/annovar
ICGC_Occurrence	International cancer genome consortium occurrence	www.openbioinformatics.org/annovar
nci60	60 human cancer cell lines	www.openbioinformatics.org/annovar
**Prediction Scores**		
CADD13_RawScore	Combined annotation dependent depletion score	https://cadd.gs.washington.edu/
CADD13_PHRED	Combined annotation dependent depletion prediction	https://cadd.gs.washington.edu/
SIFT_score	sorting intolerant from tolerant score	http://sift.bii.a-star.edu.sg/
SIFT_pred	sorting intolerant from tolerant prediction	http://sift.bii.a-star.edu.sg/
Polyphen2_HDIV_score	Pholyphen2 score based on HDIV.	http://genetics.bwh.harvard.edu/pph2/
Polyphen2_HDIV_pred	Pholyphen2 prediction based on HDIV	http://genetics.bwh.harvard.edu/pph2/
Polyphen2_HVAR_score	Polyphen2 score based on HVAR.	http://genetics.bwh.harvard.edu/pph2/
Polyphen2_HVAR_pred	Polyphen2 prediction based on HVAR.	http://genetics.bwh.harvard.edu/pph2/
LRT_score	LRT score	http://www.doclogica.com/
LRT_pred	LRT prediction	http://www.doclogica.com/
MutationTaster_score	MutationTaster score	www.mutationtaster.org
MutationTaster_pred	MutationTaster prediction.	www.mutationtaster.org
MutationAssessor_score	MutationAssessor score	http://mutationassessor.org/r3/

Annovar Name	Description	Source
MutationAssessor_pred	MutationAssessor prediction	http://mutationassessor.org/r3/
FATHMM_score	FATHMM score	*http://www.fathmm.biocompute.org.uk*
FATHMM_pred	FATHMM prediction	*http://www.fathmm.biocompute.org.uk*
PROVEAN_score	PROVEAN score	http://provean.jcvi.org/index.php
PROVEAN_pred	PROVEAN prediction	http://provean.jcvi.org/index.php
VEST3_score	VEST V3 score	*https://omictools.com/vest-tool*
CADD_raw	CADD raw score.	*https://cadd.gs.washington.edu/info*
CADD_phred	CADD phred-like score	*https://cadd.gs.washington.edu/info*
DANN_score	DANN score.	*https://omictools.com/dann-tool*
fathmm.MKL_coding_score	fathmm-MKL score for one coding variant	*http://www.fathmm.biocompute.org.uk/fathmmMKL.htm*
fathmm.MKL_coding_pred	fathmm-MKL prediction for one coding variant	*http://www.fathmm.biocompute.org.uk/fathmmMKL.htm*
MetaSVM_score	MetaSVM score.	*www.openbioinformatics.org/annovar*
MetaSVM_pred	MetaSVM prediction	*www.openbioinformatics.org/annovar*
MetaLR_score	MetaLR score	*www.openbioinformatics.org/annovar*
MetaLR_pred	MetaLR prediction	*www.openbioinformatics.org/annovar*
Eigen	Eigen	*https://eigen.tuxfamily.org/dox/*
Dann	Dann score	*https://omictools.com/dann-tool*
FATHMM_noncoding	FATHMM score for one noncoding variant	*http://www.fathmm.biocompute.org.uk*
FATHMM_coding	FATHMM score for one noncoding variant	*http://www.fathmm.biocompute.org.uk*
GWAVA_region_score	GWAVA region score	*https://www.sanger.ac.uk/sanger/StatGen_Gwava*
GWAVA_tss_score	GWAVA score	*https://www.sanger.ac.uk/sanger/StatGen_Gwava*
GWAVA_unmatched_score	GWAVA unmatched score	*https://www.sanger.ac.uk/sanger/StatGen_Gwava*
**Conservation Scores**		
integrated_fitCons_score	Fitness Consequences	*https://compgen.cshl.edu/fitCons/*
integrated_confidence_value		
GERP++_RS	GERP++ Genomic Evolutionary Rate Profiling	*https://mendel.stanford.edu/SidowLab/downloads/gerp*
phyloP7way_vertebrate	PhyloP score for 7 vertebrate species (Phylogenetic Hidden Markov Model)	*www.openbioinformatics.org/annovar*
phyloP20way_mammalian	PhyloP score for 20 mammalian species	*www.openbioinformatics.org/annovar*
phastCons7way_vertebrate	PhastCons score for 7 vertebrate species	*www.openbioinformatics.org/annovar*
phastCons20way_mammalian	PhastCons score for 20 mammalian species	*www.openbioinformatics.org/annovar*
SiPhy_29way_logOdds	SiPhy score for biased substitutions (SIte-specific PHYlogenetic)	*www.openbioinformatics.org/annovar*
