# Supplementary material for: Targeted next-generation sequencing identifies novel variants in candidate genes for Parkinson’s disease in Black South African and Nigerian patients
Source: BMC Med Genet. 2020 Feb 4;21:23. doi: 10.1186/s12881-020-0953-1 (PMC7001245; doi:10.1186/s12881-020-0953-1)
Supplement: Supplementary file 7 — Additional file 7: Figure S2. Correlation between prediction and conservation scores. [file 12881_2020_953_MOESM7_ESM.pdf]

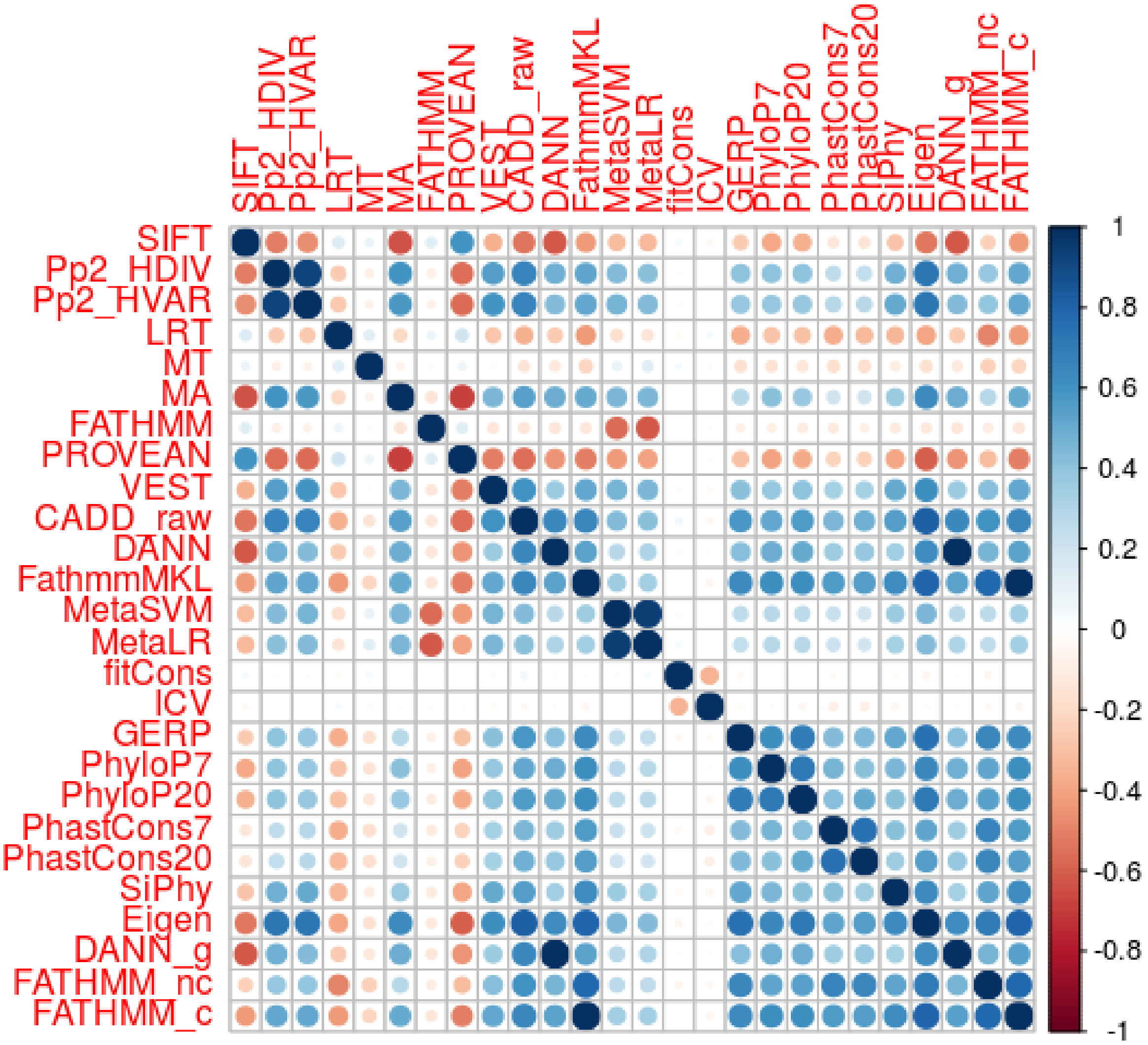

FIGURE S2. Correlation between prediction and conservation scores. We plotted the correlation matrix of 26 mutation scoring algorithm outputs for the pathogenic variants identified in this study using variant scores obtained from tNGS data. ***metaLR*** and ***metaSVM*** were well-correlated and were used to predict the deleteriousness of sequence variants. See Figure S3 for comparison of 17 different scores for each of the deleterious variants.
