# Supplementary material for: Targeted next-generation sequencing identifies novel variants in candidate genes for Parkinson’s disease in Black South African and Nigerian patients
Source: BMC Med Genet. 2020 Feb 4;21:23. doi: 10.1186/s12881-020-0953-1 (PMC7001245; doi:10.1186/s12881-020-0953-1)
Supplement: Supplementary file 9 — Additional file 9: Figure S3. Radar plots for 54 selected rare variants. [file 12881_2020_953_MOESM9_ESM.pdf]

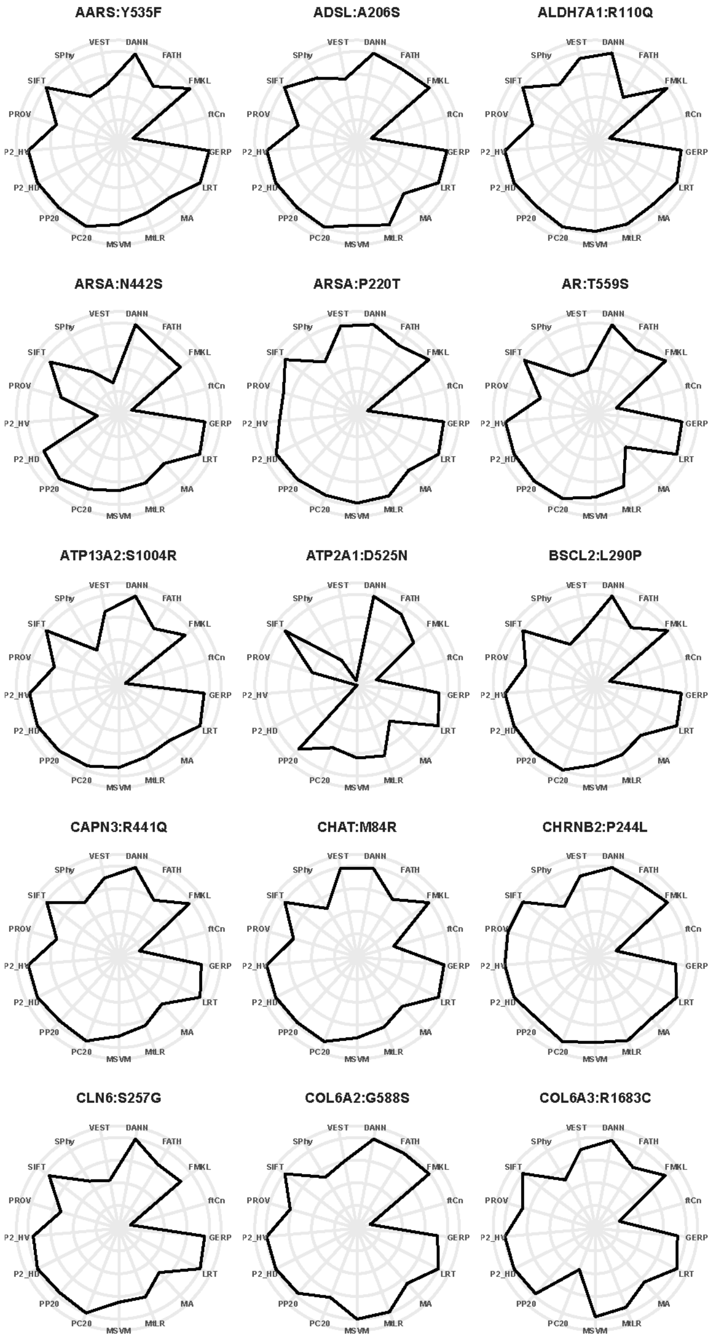

FIGURE S3: Radar plots for 54 selected rare variants. Plots are identified by symbol and amino acid change and illustrate 17 scores for each variant considered deleterious. All scores were standardized to 0–1 scale with score 1 (furthest from the center of the graph) indicating strongest evidence that the

variant is deleterious. The observations of the variants identified in the ARSA, ATP2A1, CP, DST, FLNA, GNE, PC, PSEN2, RYR1, SAMHD1, SLC12A6, and TMEM67 genes indicated limited concordance between the deleteriousness prediction tools. This figure is 4 pages.

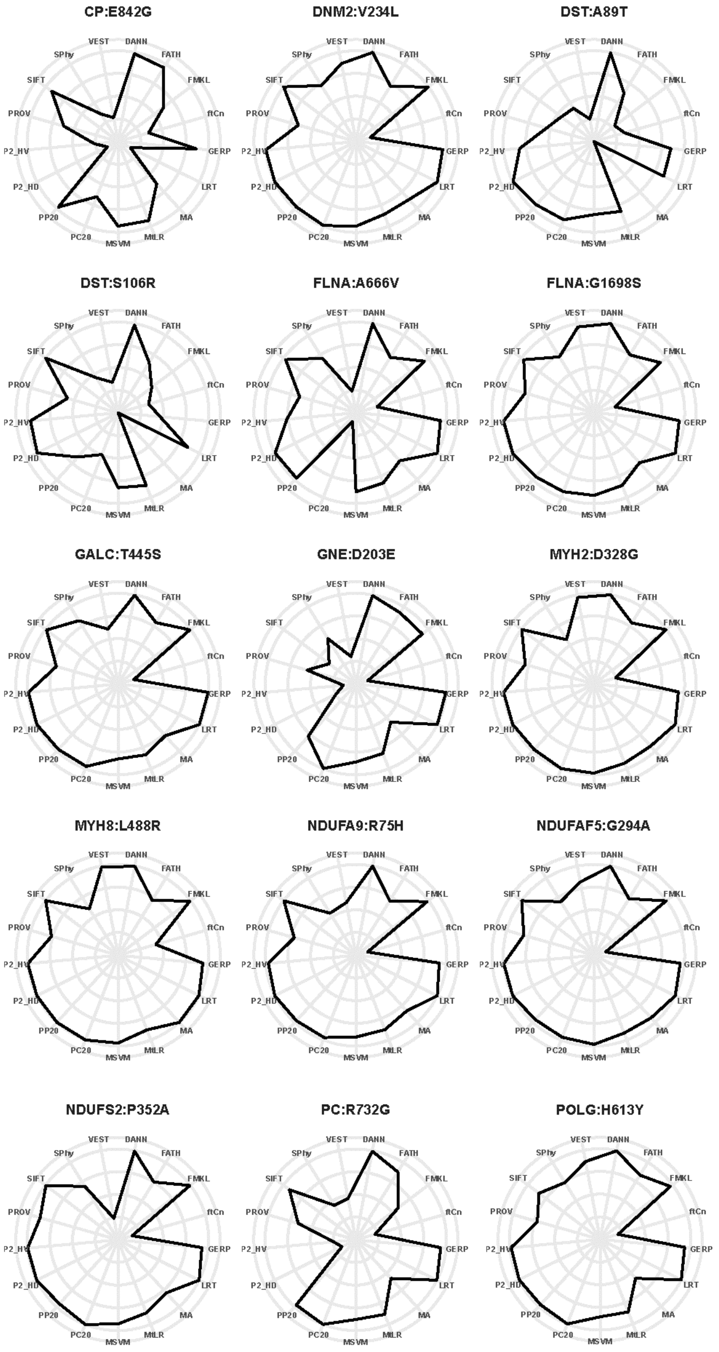

FIGURE S3 (continued)

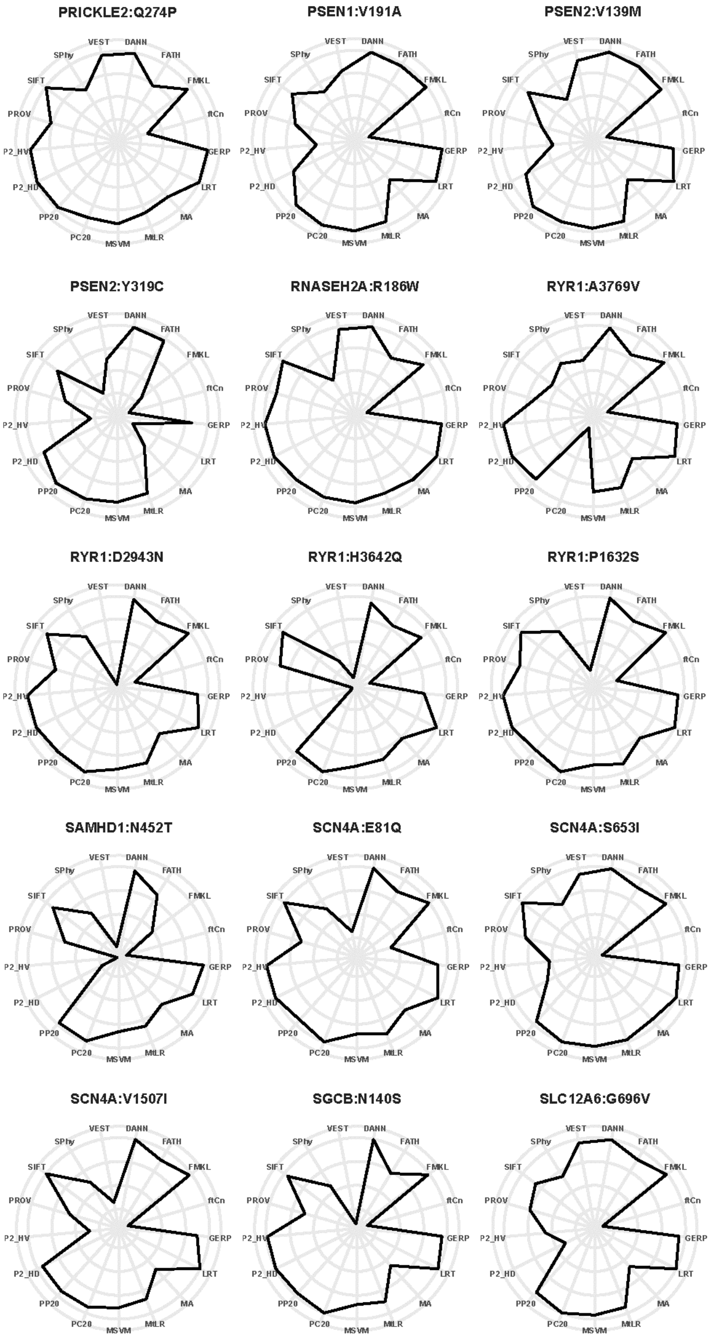

FIGURE S3 (continued)

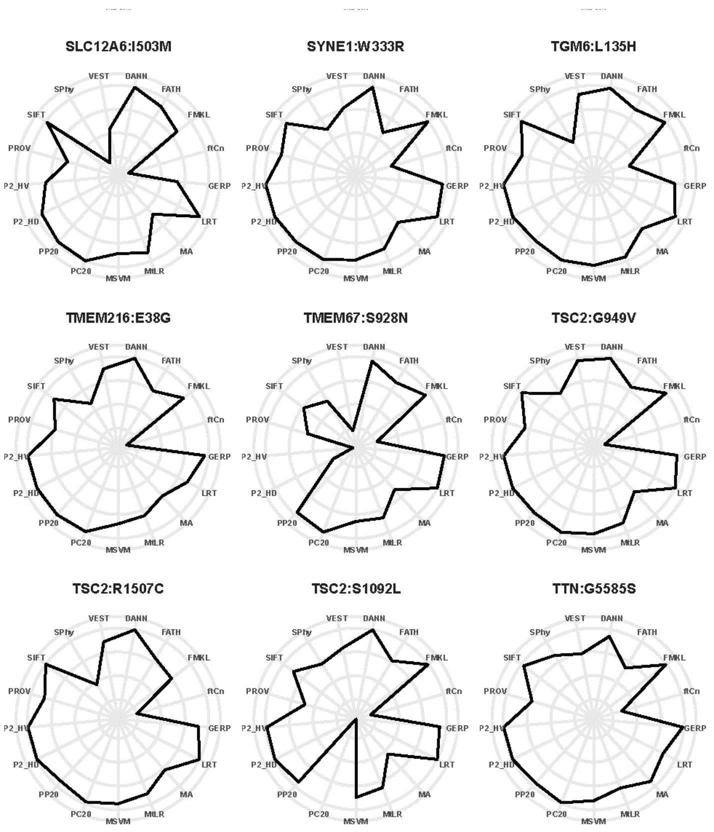

FIGURE S3 (continued)
