# Supplementary material for: Targeted next-generation sequencing identifies novel variants in candidate genes for Parkinson’s disease in Black South African and Nigerian patients
Source: BMC Med Genet. 2020 Feb 4;21:23. doi: 10.1186/s12881-020-0953-1 (PMC7001245; doi:10.1186/s12881-020-0953-1)
Supplement: Supplementary file 10 — Additional file 10: Table S6. Candidate genes with a link to Parkinson’s disease or Parkinsonism. [file 12881_2020_953_MOESM10_ESM.pdf]

Table S6: Candidate genes with a link to Parkinson's disease or Parkinsonism

Gene	Reference
ATP13A2	Yang and Xu, 2014
DST	Elliott et al., 2012
FLNA	Degos et al., 2016
GALC	Li et al., 2018
NDUFAF5	Simon et al., 2019
POLG	Davidzon et al., 2006
PSEN1	Ibanez et al., 2018
PSEN2	Ibanez et al., 2018

References

Davidzon, G., Greene, P., Mancuso, M., Klos, K.J., Ahlskog, J.E., Hirano, M., DiMauro, S., 2006. Early-onset familial parkinsonism due to POLG mutations. Ann. Neurol. 59, 859–862. https://doi.org/10.1002/ana.20831

Degos, B., Toussaint, A., Lesage, S., Brice, A., Vidailhet, M., Beldjord, C., Catala, M., 2016. PINK1 and FLNA mutations association: A role for atypical parkinsonism? Parkinsonism Relat. Disord. 26, 78–80. https://doi.org/10.1016/j.parkreldis.2016.02.023

Elliott, D.A., Kim, W.S., Gorissen, S., Halliday, G.M., Kwok, J.B.J., 2012. Leucine-rich repeat kinase 2 and alternative splicing in Parkinson's disease. Mov. Disord. 27, 1004–1011. https://doi.org/10.1002/mds.25005

Ibanez L, Dube U, Davis AA, Fernandez MV, Budde J, Cooper B, Diez-Fairen M, Ortega-Cubero S, Pastor P, Perlmutter JS, Cruchaga C, Benitez BA. Pleiotropic Effects of Variants in Dementia Genes in Parkinson Disease. Front Neurosci. 2018 Apr 10;12:230. doi: 10.3389/fnins.2018.00230. eCollection 2018. PubMed PMID:29692703; PubMed Central PMCID: PMC5902712.

Li, G., Cui, S., Du, J., Liu, J., Zhang, P., Fu, Y., He, Y., Zhou, H., Ma, J., Chen, S., 2018. Association of GALC, ZNF184, IL1R2 and ELOVL7 With Parkinson's Disease in Southern Chinese. Front. Aging Neurosci. 10. https://doi.org/10.3389/fnagi.2018.00402

Simon, M.T., Eftekharian, S.S., Stover, A.E., Osborne, A.F., Braffman, B.H., Chang, R.C., Wang, R.Y., Steenari, M.R., Tang, S., Hwu, P.W.-L., Taft, R.J., Benke, P.J., Abdenur, J.E., 2019. Novel mutations in the mitochondrial complex I assembly gene NDUFAF5 reveal heterogeneous phenotypes. Mol. Genet. Metab. 126, 53–63. https://doi.org/10.1016/j.ymgme.2018.11.001

Yang, X., Xu, Y., 2014. Mutations in the ATP13A2 Gene and Parkinsonism: A Preliminary Review [WWW Document]. BioMed Res. Int. https://doi.org/10.1155/2014/371256
