# Supplementary material for: Targeted next-generation sequencing identifies novel variants in candidate genes for Parkinson’s disease in Black South African and Nigerian patients
Source: BMC Med Genet. 2020 Feb 4;21:23. doi: 10.1186/s12881-020-0953-1 (PMC7001245; doi:10.1186/s12881-020-0953-1)
Supplement: Supplementary file 11 — Additional file 11: Figure S4. Stereoscopic crystal structure models of ATP13A2 showing the position of S1004R. [file 12881_2020_953_MOESM11_ESM.pdf]

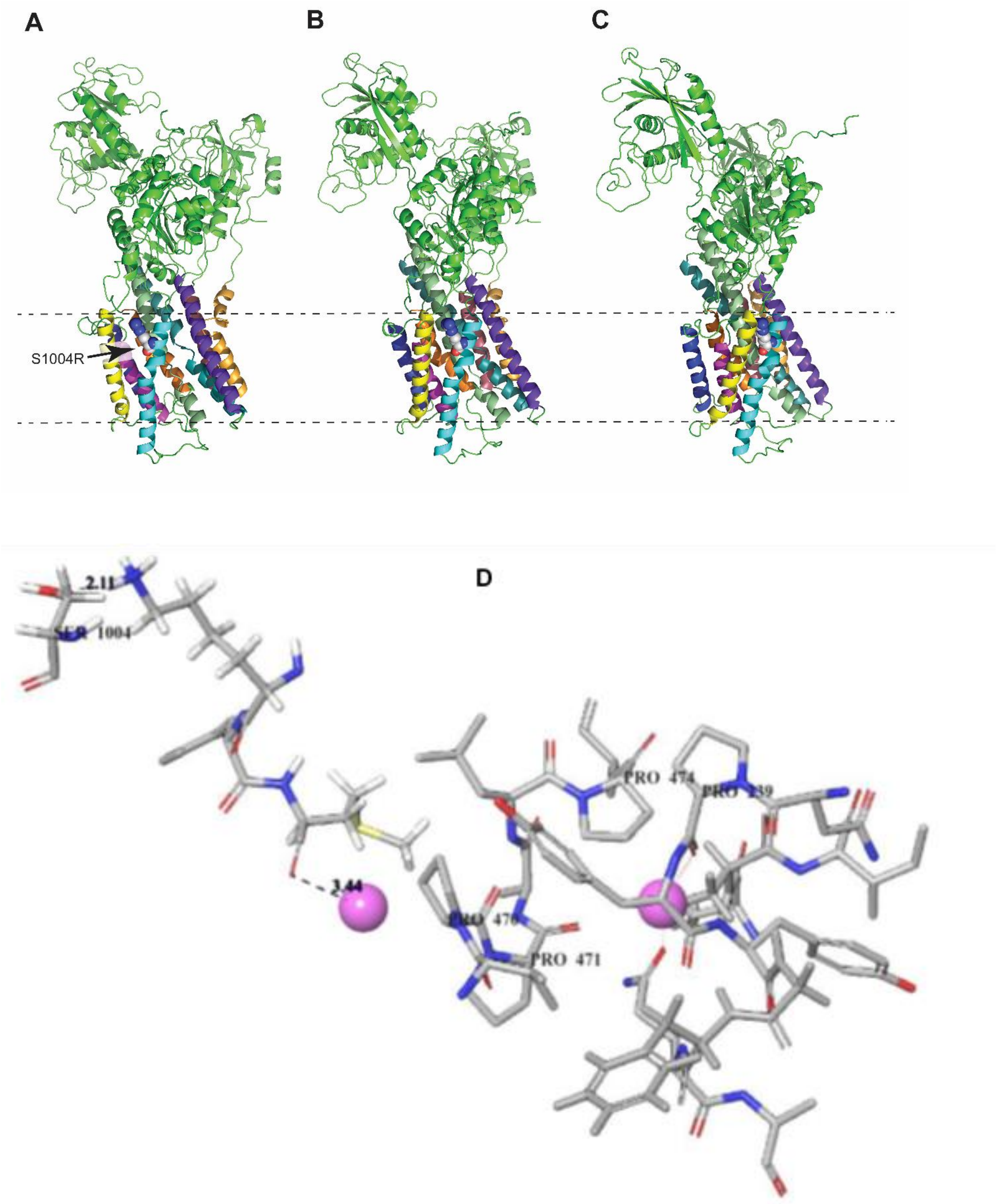
A
B
C
S1004R
D
2.11
SER 1004
3.44
PRO 474
PRO 470
PRO 471

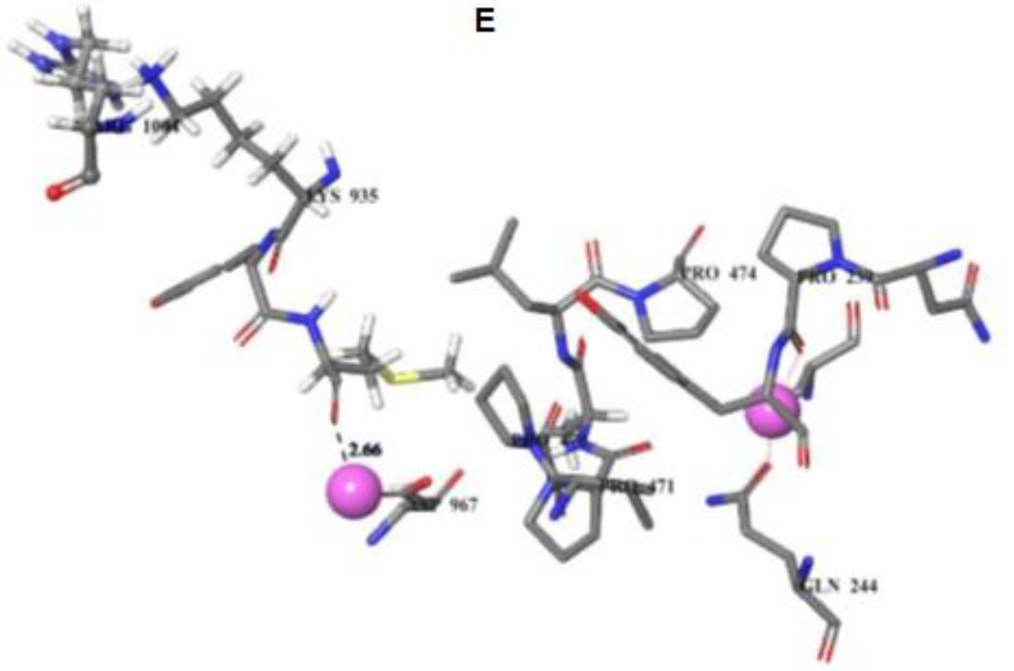

FIGURE S4: Stereoscopic crystal structure models of ATP13A2 showing the position of S1004R.

The structure of ATP13A2 was modelled by submitting the 1180 amino acid UniProt accession ACQ9NQ11 to the Phyre2 server. In brief, the HHblits algorithm identified 366 homologous sequences from a database where no two pairs of sequence share >20% identity. The sequences were used to generate a “sequence profile” and the secondary structure for each sequence was predicted using PSIPRED. The profile and secondary structures were used to compute a hidden Markov model (HMM) which was compared to a database of precomputed HMMs for known crystal structures. Six known crystal structures (c2zxeA, c3b9bA, c3ixzA, c3b8eC, c1mhsA, and c6a69A) were used to constrain portions of the predicted structure for ATP13A2. The six had confidence scores of 100, and structural alignment of 817 to 892 residues. Combined, they represented 954 of the 1180 residues. The structure of the remaining 226 amino acids was then predicted *ab initio*. The N-terminus contained 178 of the 226 residues for which there was no homologous structure. The remaining residues were in short loops or coils between constrained structures.

(A) Structure of ATP13A2 in ribbon format with arginine (S1004R) shown with spheres in CPK atom coloring and identified with text and an arrow. The structure is rotated **(B)** 22.5º and **(C)** 45º along the Y axis. The dotted lines represent the approximate position of the endosomal phospholipid bilayer. The transmembrane helices, M1 to M10, are color coded: bright orange, M1 (aa 228 – 254); raspberry red, M2 (aa 257 – 277); purple blue M3 (aa 420 – 448); deep teal, M4 (aa 454 –491); pale green, M5 (aa 913 – 952); orange, M6 (aa 961 – 980); cyan, M7 (aa 997 – 1026); magenta, M8 (aa 1046 – 1064); blue, M9 (aa1079 – 1097); and yellow, M10 (aa 1116 – 1140).

Protein modelling are shown for **(D)** S1004 and **(E)** R1004.
